# Sixth Nerve Palsy from Cholesterol Granuloma of the Petrous Apex

**DOI:** 10.3389/fneur.2017.00048

**Published:** 2017-02-15

**Authors:** Ségolène Roemer, Philippe Maeder, Roy Thomas Daniel, Aki Kawasaki

**Affiliations:** ^1^Department of Ophthalmology, University of Lausanne, Jules-Gonin Eye Hospital Fondation Asile des aveugles, Lausanne, Switzerland; ^2^Department of Radiology, University of Lausanne, Lausanne University Hospital, Lausanne, Switzerland; ^3^Department of Clinical Neurosciences, Section of Neurosurgery, University of Lausanne, Lausanne University Hospital, Lausanne, Switzerland

**Keywords:** sixth nerve palsy, abducens palsy, diplopia, esotropia, cholesterol granuloma, skull-base tumor, petrous apex tumor

## Abstract

Herein, we report a patient who had an isolated sixth nerve palsy due to a petrous apex cholesterol granuloma. The sixth nerve palsy appeared acutely and then spontaneously resolved over several months, initially suggesting a microvascular origin of the palsy. Subsequent recurrences of the palsy indicated a different pathophysiologic etiology and MRI revealed the lesion at the petrous apex. Surgical resection improved the compressive effect of the lesion at Dorello’s canal and clinical improvement was observed. A relapsing–remitting sixth nerve palsy is an unusual presentation of this rare lesion.

## Introduction

### Case Report: Presentation

A healthy 49-year-old man first noted painless horizontal diplopia in October 2015 and presented to the emergency room. Visual testing including acuity, color vision, and visual field was normal in both eyes. There was a small anisocoria in dim light with the right pupil measuring 6.5 mm and the left pupil 6.0 mm. Topical apraclonidine did not show any evidence of sympathetic denervation hypersensitivity. Ocular motor examination revealed an esotropia and limited abduction of the right eye. The abducting saccade was also noted to be slow. Facial sensory and motor functions were intact. Hearing testing by finger rustle was symmetric. The examination findings were consistent with a diagnosis of right sixth nerve palsy and physiologic anisocoria.

Blood tests including glucose, full blood count, sedimentation rate, C-reactive protein, acetylcholine receptor antibodies, antineutrophil cytoplasmic antibody, angiotensin-converting enzyme, anti-GQ1b antibodies, and brain imaging were performed. A CT scan was said to be normal. A head MRI reportedly showed an asymmetry between the right and left cavernous sinus but a specific diagnosis was not proposed. A 2-week treatment trial with pyridostigmine did not improve his condition.

Thereafter over the next several weeks, his esotropia was noted to improve spontaneously and gradually. We first saw the patient in late December 2015 at which time the sixth nerve palsy was almost completely resolved. Up to this point, the clinical course seemed consistent with a microvascular sixth nerve palsy.

In early 2016, the patient developed a non-specific viral syndrome with mild vertigo and fever, and then experienced acute recurrence of the right abduction deficit with diplopia. This recurrence was associated with mild periorbital pain on the right side and diffused facial tingling. Repeat clinical examination of the cranial nerves, in particular V, VII, and VIII, did not reveal any deficits. The symptoms resolved spontaneously within a few days.

**Figure 1:**
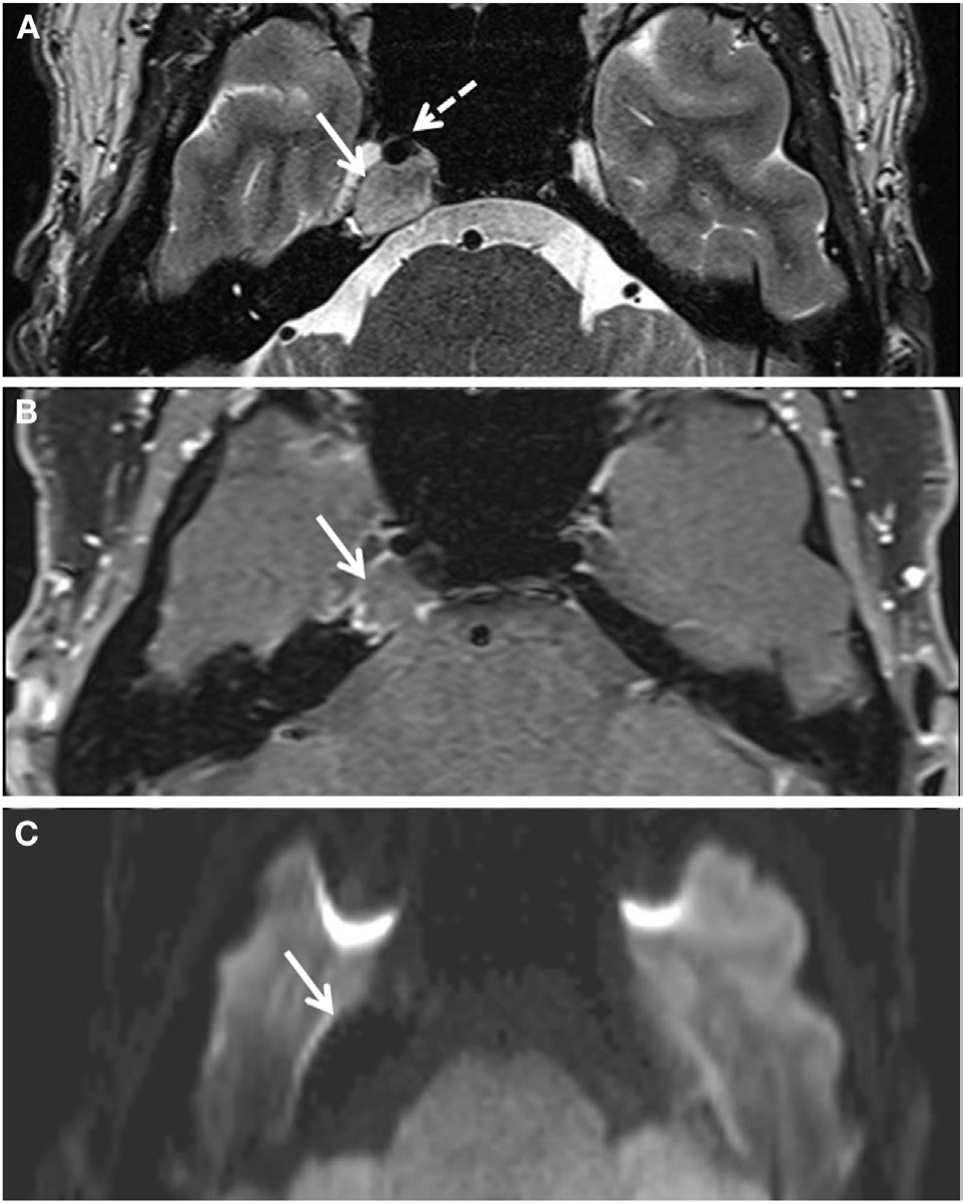
**Lesion (white solid arrows) of the right petrous apex as viewed on different axial MRI sequences**. **(A)** T2-weighted imaging shows a slightly heterogeneous lesion with parts isointense to white or gray matter. The dotted arrow shows the internal carotid artery. **(B)** The lesion is slightly hypointense to brain on T1 VIBE image and demonstrates slight peripheral enhancement following injection of gadolinium contrast. **(C)** The lesion is hypointense on diffusion trace image.

**Figure 2:**
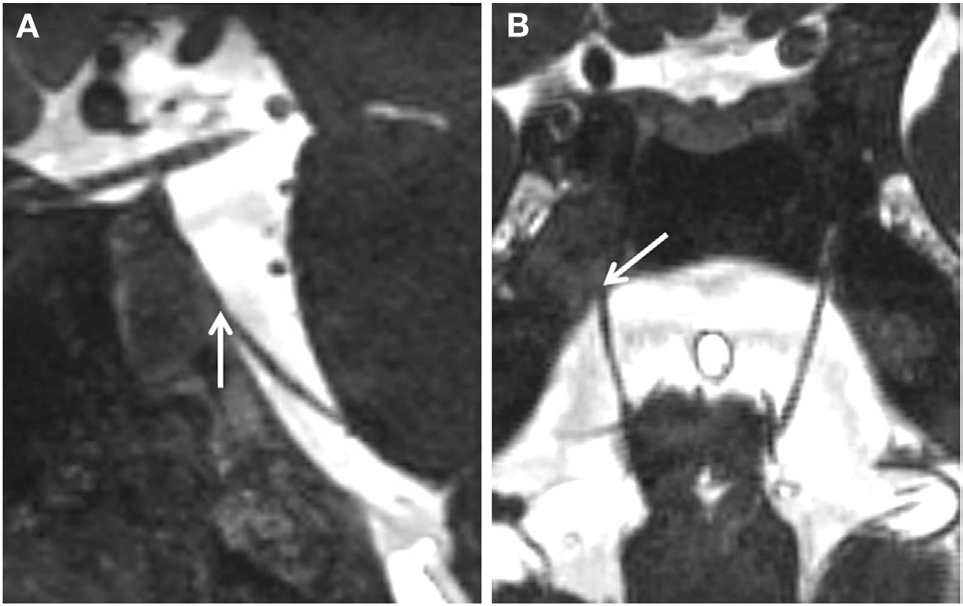
**Close-up view (A) sagittal and (B) axial of the region of Dorello’s canal**. The lesion is seen to enter Dorello’s canal (white arrows) and compress the right sixth nerve.

**Figure 3:**
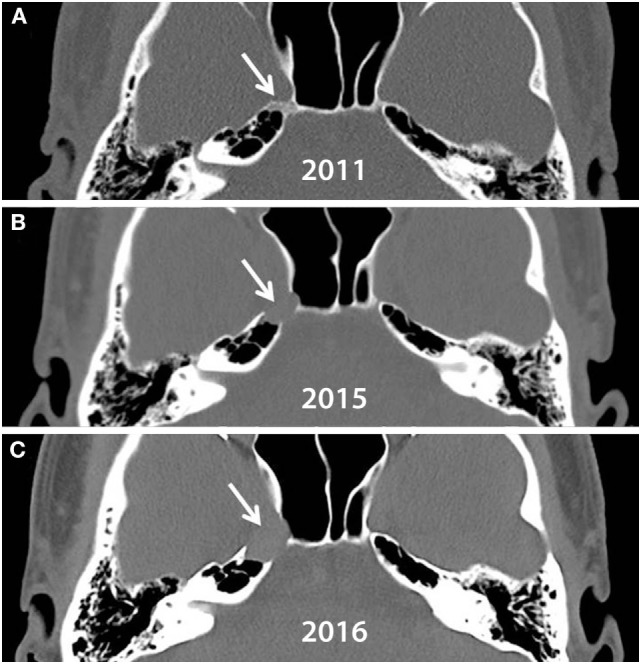
**Axial view of head CTs taken in (A) 2011, (B) 2015, and (C) 2016**. A small lesion (arrow) causing mild bony abnormality of right petrous apex is visible on 2011. Both petrous apices are highly pneumatized. The lesion progressively erodes the right petrous apex over 5 years.

Because of this atypical course, an MRI was repeated and showed a small lesion at the right petrous apex. The lesion was hypointense to brain on T1-weighted and diffusion trace imaging and slightly heterogeneous on T2-weighted images (Figure 1). There was slight peripheral enhancement. The lesion appeared to abut the internal carotid artery and compress the right sixth nerve at Dorello’s canal (Figure 2). At this time, we requested to have all the patient’s previous imaging studies, including a head CT which had been performed in 2011 following a cycling accident. A careful retrospective review of sequential CT scans from 2011 to 2016 showed a petrous apex lesion in 2011, which increased in size and progressively eroded the right petrous bone over the next 5 years (Figure 3). Additionally, the petrous bone was noted to be extensively pneumatized. The radiologic features were felt to be most consistent with a cholesterol granuloma (CG) of the petrous apex.

## Background

Cholesterol granulomas are encapsulated cystic lesions that arise from blood by-products and foreign body reaction to cholesterol crystals. Initially described in the peritoneum, CGs may arise in many locations, including the region of the middle ear, mastoid, and petrous apex (1).

The pathogenesis of a CG is yet debated. The original hypothesis, often termed the obstruction-vacuum theory (2, 3), proposes that the inciting event is mucosal swelling which obstructs air cell outflow tracts in the petrous apex. As trapped gas is resorbed, the negative pressure in the unventilated spaced creates a vacuum into which blood exudes. The blood products, including cholesterol, trigger a foreign body reaction, ultimately resulting in a granuloma. Such a pathophysiologic mechanism would expect to result in CGs more commonly among patients with recurrent otitis media with effusion, a common condition causing chronic hypoventilation and aeration in surrounding air cells. Yet CG is rare lesion in this population. Another seeming paradox is that pneumatization, hence aeration, of the petrous bone is so common in CGs arising in this region that is considered a radiographic feature of the diagnosis. Other observations difficult to reconcile with this theory are the intralesional hemorrhage and blood seepage leading to cyst expansion (4).

A newer theory has thus emerged and is termed the exposed marrow hypothesis. It posits that there is an underlying pathologic communication between the mucosa of the air cells of the petrous apex and the petrous marrow, a highly vascularized structure. This interface leads to a predilection for hemorrhage into air cells. The hemorrhage may occur spontaneously but the hypothesis suggests that it is more likely triggered by an external event such as head trauma, barometric stress as in air travel, a hypertensive episode, a strong Valsalva maneuver or even an upper respiratory tract infection. This hypothesis maintains that “cholesterol granulomas both arise from and are sustained by anatomic contiguity to a blood source” (4). Compression of the feeding blood vessels induces an inconstant blood product release and lack of drainage allows stagnation of hemorrhagic contents. Breakdown of blood cells leads to the formation and accumulation of cholesterol crystals and these crystals incite the irreversible granulomatous reaction (5). As such, CGs are often a collection of viscous material which can slowly expand with time.

Cholesterol granulomas may be asymptomatic or may cause symptoms related to compression of neighboring structures. In a series of 90 patients with a CG of the petrous apex, the average age at presentation was 43.1 years (median 42.0, range 8.0–77.0 years) (6). All were unilateral lesions. Over half of patients (56.7%) had headache and one-third had dizziness (35.6%). Other symptoms were facial paresthesia or pain (12.2%), sensorineural hearing loss (6.7%), facial palsy (2.2%), and a single patient (1.1%) complained of diplopia. An earlier review of the literature of 92 cases reported a much higher rate of hearing loss (37%), but diplopia was still infrequent, reported in 5.4% of patients (7).

In these large series, it appears that diplopia is a relatively rare manifestation of a CG of the petrous apex (1–5%) (6, 7). In other studies with smaller cohorts, the prevalence of diplopia is highly variable, ranging from 0 to 43%, and the diplopia is often accompanied by other symptoms or signs (8–10). Our patient’s initial clinical presentation is thus unusual in that he had a painless diplopia from a unilateral and isolated sixth nerve palsy. The spontaneous remission of the sixth nerve palsy following initial presentation added to the initial diagnostic confusion regarding the cause of the sixth nerve palsy.

## Discussion

### Case Report: Follow-up and Treatment

In this patient, the radiologic features of the lesion were felt to be most consistent with a CG of the petrous apex. Because of its proximity to the carotid artery, observational management was recommended. The patient’s diplopia and abduction deficit again improved spontaneously over the following weeks and then stabilized, leaving a residual minor esotropia which was symptomatically alleviated with prismatic correction. The patient was then doing well and returned to all activities of daily living.

Six months later, the patient suddenly experienced an acute and severe worsening of diplopia. Examination revealed complete absence of abduction of the right eye. An MRI showed a definite expansion of the lesion (15 mm × 14 mm × 12 mm) compared to the lesion (13 mm × 12 mm × 9 mm) seen on MRI 6 months earlier. No acute hemorrhage within the CG was visible. Surgical approach to the lesion was achieved through a trans-sphenoidal access. The lesion contained a thick brownish liquid that was removed completely. The cyst wall was curetted out, and the cavity was left with a wide opening into the sphenoid sinus in order to ensure that the cyst contents drained into the sinus in case the cyst contents reformed. The few small fragments of the cyst wall that was available for histopathology showed only collagenous fragments.

**Figure 4:**
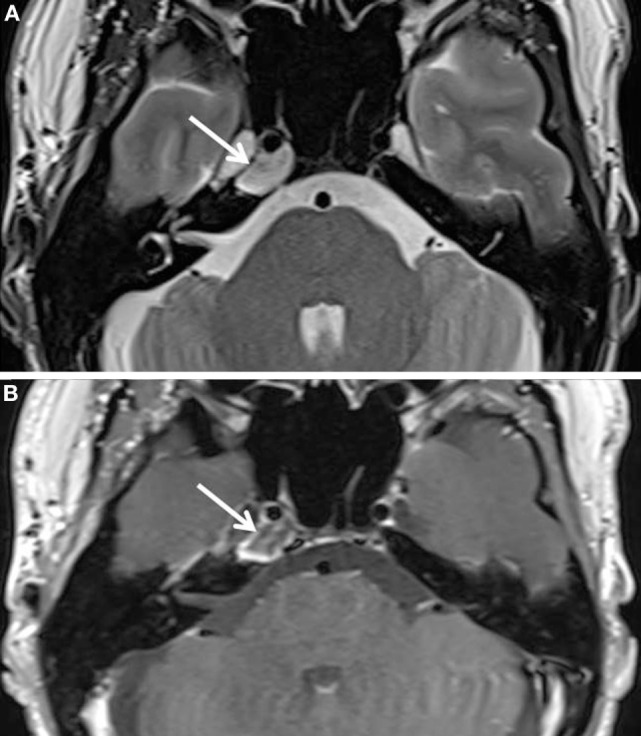
**Postoperative MRI images**. **(A)** T2-weighted axial and **(B)** T1-weighted axial images after injection of gadolinium **(B)** show a notable regression of the right petrous apex lesion (white arrows). The content of the lesion is still heterogeneous. There is slight peripheral enhancement.

One month postoperatively, the patient’s sixth nerve palsy was significantly improved, and 3 months postoperatively, it was almost completely resolved. The patient has been stable for 6 months postoperatively, showing no significant abduction defect and a minor esodeviation of two diopters in primary position. The postoperative MRI confirmed the reduction in the size of the cystic formation at the petrous apex (Figure 4).

### Case Report: Comments

Cholesterol granulomas are typically slow-growing benign lesions. Histopathologically, they contain thick, brown mucoid fluid, multinucleated giant cells surrounding cholesterol crystals in the submucosa or fibrous connective tissue, pathologic blood vessels, hemosiderin-laden macrophages, and round cells (5). One study found that 86% (76 of 90 cases) of petrous apex CGs have remained radiographically and clinically stable for an average of 46 months (6).

Cholesterol granulomas at the petrous apex typically cause headache and hearing loss. Our patient’s lesion caused an isolated sixth nerve palsy. Typically, a structural lesion at the skull base which compresses the sixth nerve palsy causes a slowly progressive deficit but our patient’s palsy demonstrated a remitting and relapsing course during the 7 months following onset. As mentioned earlier, the spontaneous resolution of the sixth nerve palsy in the first 2 months following onset led to initial diagnostic confusion as such a clinical course is more suggestive of a microvascular origin.

Microvascular sixth nerve palsy is an entity that typically affects older adults who have one or more vascular risk factors. These palsies are presumably due to insufficient perfusion of the peripheral portion of the nerve which causes secondary demyelination. Most (86%) microvascular sixth nerve palsies recover spontaneously within 3 months (11). For this reason, many clinicians forego imaging of an acute and isolated sixth nerve palsy in adults over age 50 years, particular if vascular risk factors are present (12). Recent studies using MRI, however, have demonstrated a higher yield of lesions associated with sixth nerve palsy in this population than previously reported (13).

What might be the reasons for such a remitting and relapsing course of a sixth nerve palsy associated with CG? In our patient, the granuloma was in contact with the internal carotid artery at the skull base (Figure 2). We can conjecture that remodeling of the CG, as explained by the exposed marrow hypothesis, may alter local hemodynamics and precipitate a relative perfusion deficit to the sixth nerve, causing palsy. In this scenario, the clinical evolution would be expected to be similar to a *de novo* microvascular nerve palsy (12, 14).

When the patient experienced an acute and severe relapse of the nerve palsy, we wonder if a sudden and significant expansion of the CG resulted in direct compression of the sixth nerve at Dorello’s canal, adding a second insult to that of hypoperfusion. This conjecture is supported by a visible increase in the size of the patient’s CG compared to a scan 6 months previously (Figure 3). Such a compressive etiology for the acute nerve palsy would also be consistent with the rapid recovery of the sixth nerve palsy after decompressive surgery.

Other skull base lesions have been previously reported in association with a sixth nerve palsy. These include chordoma, metastasis, meningioma, chondrosarcoma, aneurysm, pituitary macroadenoma, schwannoma, and glioma (12, 13, 15). In most cases, a structural lesion at the skull base results in isolated or combined cranial nerves palsies having a slowly progressive course (16). In one series of seven patients with sixth nerve palsy caused by skull base lesions which spontaneously remitted, the authors postulated that mechanisms for such recovery may relate to remyelination, restoration of axoplasmic flow, or improved perfusion. Such mechanisms may also underlie the remitting phase of our patient’s sixth nerve palsy (15).

## Concluding Remarks

The diagnosis of CG remains challenging because of the rarity of this lesion and because of the different clinical manifestations, which depend in part on the location of the granuloma. This is a case report of a petrous apex CG which presented as an isolated sixth nerve palsy which is an unusual manifestation of this skull base lesion. In addition, a remitting and relapsing course of the sixth nerve palsy in our patient is atypical for a skull base lesion, particularly as CGs tend to be slowly and progressively expansive. This case highlights the need to consider neuroimaging for isolated sixth nerve palsy, particularly in patients without vascular risk factors, as it demonstrates how a compressive lesion may cause a palsy that spontaneously remits.

## Author Contributions

SR, PM, RD, and AK: substantial contributions to the conception or design of the work, or the acquisition, analysis, or interpretation of data for the work; drafting the work or revising it critically for important intellectual content; final approval of the version to be published; agreement to be accountable for all aspects of the work in ensuring that questions related to the accuracy or integrity of any part of the work are appropriately investigated and resolved.

## Conflict of Interest Statement

AK has received book royalties from Cambridge University Press. SR, PM, and RD have nothing to disclose. The reviewer JJ and handling editor declared their shared affiliation, and the handling editor states that the process nevertheless met the standards of a fair and objective review.
